# Red cell transfusion in the critically ill patient

**DOI:** 10.1186/2197-425X-3-S1-A915

**Published:** 2015-10-01

**Authors:** L Kersan, M McGarvey, C MacNeil, A Mackay, K McDowall, R Ahmed

**Affiliations:** Victoria Infirmary, Glasgow, United Kingdom; Queen Elizabeth University Hospital, Intensive Care Unit, Glasgow, United States Minor Outlying Islands

## Introduction

It has been more than a decade since the TRICC trial established the concept of restrictive transfusion. The CRIT study subsequently showed that RBC transfusion may be an independent predictor of worse outcome. We undertook an audit to determine how our practice compares to the current evidence.

## Methods

All ICU patients in the Victoria Infirmary receiving red blood cells between January 2013 and January 2014 were identified. The haemoglobin transfusion trigger was obtained from our clinical information system. The presence of bleeding, severe sepsis, ischaemic heart disease and traumatic brain injury were determined from our intensive care patient database and transfusion request forms.

## Results

Red blood cells were issued a total of 124 times during the study period. In only 15 (12%) instances was the Hb trigger < 70g/L. The remaining 109 (88%) units transfused were at a Hb >70g/L. Within this high transfusion trigger group, there were 52 (48%) cases of bleeding, 67 (61%) cases of severe sepsis, 28 (26%) cases of ischaemic heart disease and 2 (2%) cases of traumatic brain injury. This left only 15 (12%) occasions where there appeared to be no clear justification for the decision to transfuse.

## Conclusions

Despite the well recognized Hb trigger of 70g/L, there has been ambiguity regarding transfusion triggers in certain patients. New evidence is emerging to support restrictive transfusion in acute upper GI bleeding [1], septic shock [2] and traumatic brain injury [3]. Only 12% in our audit had no clear justification for transfusion but it is arguable that a restrictive strategy should have been used in these subgroups. Of note, 19 cases of severe sepsis also had coexisting coronary artery disease as did 8 cases of bleeding which were not limited to upper GI bleeding. In addition much of the evidence is so recent that it predates our audit but there is certainly a need for increased awareness of these new trials.
